# Moving to the Outskirts: Interplay Between Regulatory T Cells and Peripheral Tissues

**DOI:** 10.3389/fimmu.2022.864628

**Published:** 2022-04-29

**Authors:** Anna Estrada Brull, Camilla Panetti, Nicole Joller

**Affiliations:** Department of Quantitative Biomedicine, University of Zurich, Zurich, Switzerland

**Keywords:** regulatory T cells, homing, tissue homeostasis, tissue repair, immune regulation

## Abstract

Regulatory T cells (Tregs) restrain excessive immune responses and dampen inflammation. In addition to this classical immune suppressive role, Tregs in non-lymphoid tissues also promote tissue homeostasis, regeneration and repair. In this review, we outline our current understanding of how Tregs migrate to peripheral tissues and the factors required for their maintenance at these sites. We discuss the tissue-specific adaptations of Tregs at barrier and immuno-privileged sites and the mechanisms that regulate their function within these organs. Furthermore, we outline what is known about the interactions of Tregs with non-immune cells in the different peripheral tissues at steady state and upon challenge or tissue damage. A thorough understanding of the tissue-specific adaptations and functions of Tregs will potentially pave the way for therapeutic approaches targeting their regenerative role.

## Introduction

Regulatory T cells (Tregs) are essential for suppressing autoreactive immune responses and controlling the effector immune response to prevent autoimmunity and maintain tissue homeostasis. Tregs are defined by their expression of the transcription factor Foxp3, which confers them with their suppressive function (1). Furthermore, they are marked by constitutive expression of the co-inhibitory receptor CTLA-4 and the high affinity IL-2 receptor α-chain CD25 and strictly depend on extrinsic IL-2 for their survival (2–4). Tregs use a wide array of mechanisms to exert their suppressive activity, including the secretion of inhibitory cytokines, such as IL-10 and TGF-β (5, 6), the modulation of antigen presenting cells (APCs) (7), direct cytolysis (8) as well as metabolic disruption of the target effector cells by cytokine sequestration (8–10). In addition, non-canonical functions, including a direct role in tissue protection and repair, have recently been attributed to Tregs (11, 12). Tregs thus cover a broad functional spectrum and represent a heterogenous population composed of specialized subsets that fulfill these diverse functions.

To exert their tissue protective functions, Tregs home to and localize within the peripheral tissue niche, where they are embedded in a tissue-specific network of immune and non-immune cells. This cellular network constantly interacts with and shapes the Treg population in the respective tissue, while conversely the Tregs shape the tissue niche. In this review, we will outline how Tregs seed the peripheral tissues and highlight the importance of this process for exerting their effector functions. We will further discuss the tissue-specific adaptations of Tregs as well as their functions at barrier and immuno-privileged sites at steady state and upon challenge or tissue damage.

## Treg Homing

**Figure 1:**
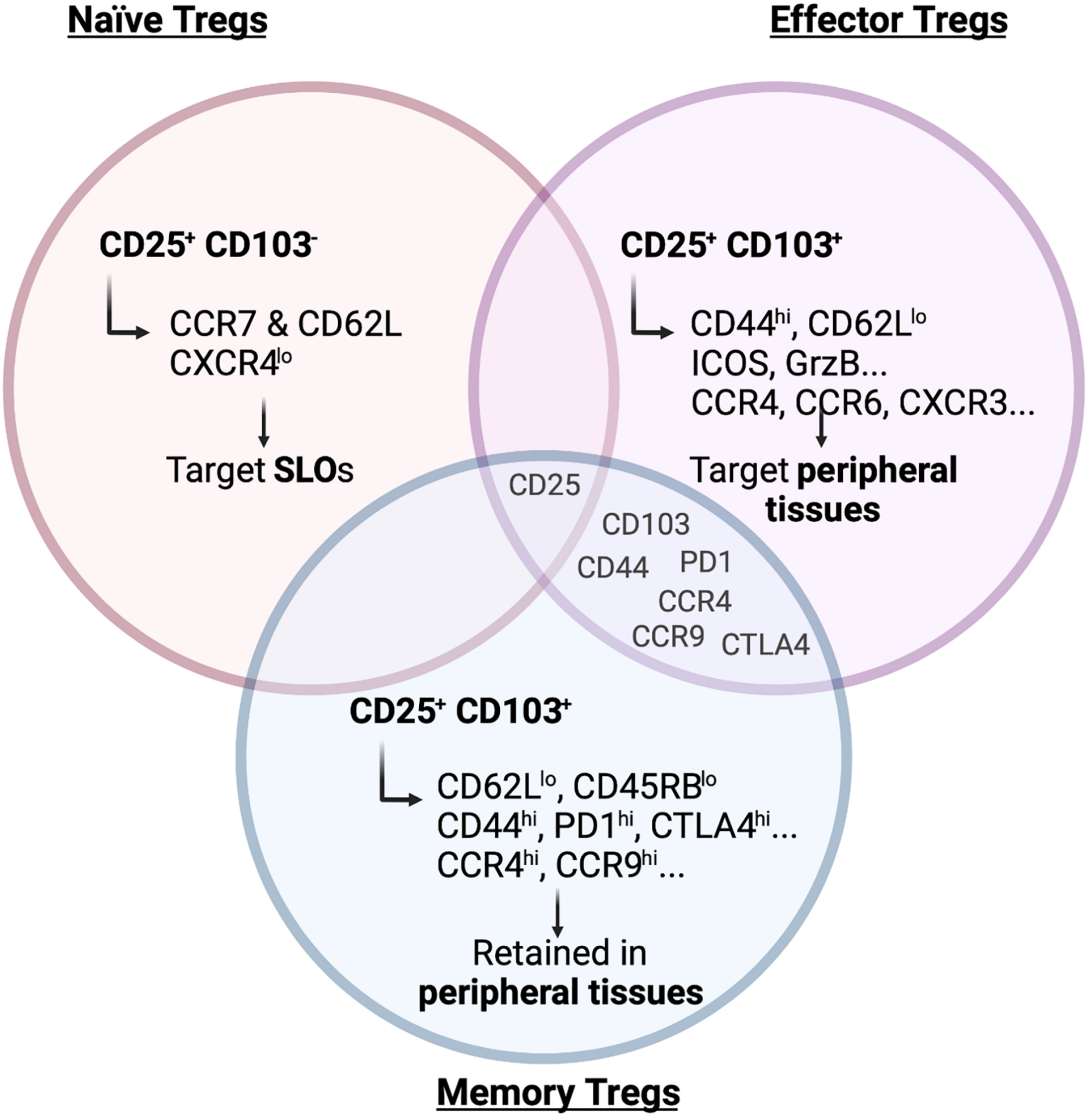
Main markers of naïve, effector and memory Tregs. Surface receptors expressed on Tregs are crucial to determine their migration and residency in peripheral tissues. As depicted in the figure, markers of effector and memory Tregs are to a large degree shared but naïve Tregs show a clearly distinct marker profile. Illustration created with BioRender.com.

Already 20 years ago it became clear that Tregs show a high degree of heterogeneity regarding their localization and function, when Lehmann and colleagues subdivided the Treg population based on its expression of CD103, the integrin a_E_ (13). Integrin a_E_b_7_ binds to E-cadherin, expressed on epithelial cells but not on the endothelium, and was therefore used as a marker for intraepithelial T cells residing in non-lymphoid tissues. Lehmann et al. found that CD103^+^ Tregs express higher levels of the co-inhibitory receptor CTLA-4, which confers them with a higher suppressive capacity. Subsequent studies additionally used CD25 to further segregate Tregs into phenotypically and functionally distinct subsets. CD103^-^CD25^+^ Tregs are enriched for CCR7 and CD62L (L-selectin) and therefore show a naïve phenotype, whereas CD103^+^ Tregs express CD44, ICOS, Granzyme B (GrzB), Integrin b_1_ and several chemokine receptors indicating a more active, effector-like phenotype (14) (**Figure 1**). Other groups used the expression of CD44 and CD62L to divide the Treg population into central Tregs (CD44^lo^CD62L^hi^) and effector-like Tregs (CD44^hi^ CD62L^lo^) (15). These Treg subsets not only differ in their activation status but also in their migratory capacity. Although both the CD103^+^ CD25^+^ double-positive and the CD25^+^ single-positive Tregs migrate towards the CCR7 ligand CCL21, CD103^+^ Tregs show a higher infiltration to sites of active immune responses by migrating towards CCL17 (CCR4 ligand), CCL20 (CCR6 ligand) and CXCL9 (CXCR3 ligand), which are released upon inflammation (14). In fact, only the CD103^+^ Tregs can effectively and specifically infiltrate the inflamed skin after hapten 2,4-dinitrofluorobenzene (DNFB) treatment in mice (14), a murine model of contact hypersensitivity. Similarly, only CD103^+^ Tregs constrain the development of intestinal inflammation after transfer of CD4^+^ T cells into SCID mice (13). This observation suggested that primarily the CD103^+^ Tregs react to inflammatory stimuli present at peripheral tissues, while CD25^+^ Tregs respond to chemokines present in lymphoid organs, allowing for a distinction between naïve CD103^-^CD25^+^ and effector/memory-like CD103^+^CD25^-^ Tregs.

Having defined the concept of naïve and effector-like Tregs, Siegmund and colleagues later reported that effector T cell activation and differentiation in the lymph nodes is regulated by CD103^-^ CD25^+^ Tregs (16). This population is more homogeneous in its expression of chemokine and homing receptors and is found recirculating between blood and lymph nodes, whereas the effector/memory Treg population shows a more heterogeneous pattern reflecting its ability to migrate to different tissues (17). Nevertheless, these are not two different Treg populations originating from a common progenitor, but rather they represent distinct differentiation stages. The T cell maturation process naturally occurring in the thymus gives rise to a homogeneous CD62L^+^CCR7^+^CXCR4^lo^ Treg population targeted preferentially to secondary lymphoid organs (SLOs) (18) (**Figure 1**). Upon antigen-specific priming within the lymphoid organs the expression pattern of homing and chemokine receptors is then altered (18). During the priming phase, on day 3 after immunization, approximately 60% of the Tregs localized in the lymph node lose the expression of CCR7 and CXCR4. In return, they gain CXCR3, CCR4, CCR6 and/or CCR8 expression, allowing them to migrate to peripheral tissues (18). Interestingly, this shift in the chemokine receptors expression pattern in Tregs is more pronounced than the corresponding changes in conventional Foxp3^-^ T cells and also precedes them. This differential expression pattern may represent a mechanism to ensure timely and efficient recruitment of Tregs to the peripheral sites to help maintain tissue integrity. Indeed, Tregs already seed tissues during development and the early post-natal periods. Importantly, their depletion during this time period can cause alterations in tissue structure (19), suggesting that they play an active role in shaping peripheral tissues.

Besides the acquisition of homing receptors in lymph nodes, recent studies showed that also the tissue phenotype of Tregs is primed in SLOs. This phenomenon was studied in the context of visceral adipose tissue (VAT) Tregs, which express the VAT Treg-specific transcription factor PPARγ (20). As they arise in the thymus, Tregs are PPARγ^-^, but within the SLOs a small Treg population with intermediate PPARγ expression emerges (21, 22). These Tregs already express some aspects of the VAT Treg signature but only become PPARγ^hi^ and acquire the full VAT Treg phenotype and epigenetic profile once they enter the VAT (21, 22). This two-step differentiation process was also apparent in a study that analyzed transcriptional trajectories of single Treg cells as they progress from SLOs to non-lymphoid tissues (23). A subset of Tregs in the SLOs already displays tissue-like transcriptional profiles, representing a portion of the tissue-specific transcriptional programs as well as the chemokine receptors that allow for homing to the respective tissues (23). Moreover, these intermediate differentiation stages of tissue Tregs are shared between different Treg subsets, regardless of their terminal tissue (22, 23). Further characterization of these tissue Treg precursors revealed that they depend on the transcription factor Batf for their development and, when present in the SLOs, express Nfil3 (24). Furthermore, acquisition of a tissue Treg phenotype goes along with the loss of the transcription factor ID3 (25). The target tissue of these transitional Treg subsets is determined by their antigen specificity and hence their T cell receptor (TCR) (21). As a consequence, Tregs seeding the VAT, skin or colon represent clonally expanded populations that are also represented in smaller numbers in the lymph nodes, where they are primed before migrating to the peripheral tissue and acquiring their tissue phenotype (21, 24, 26). Single-cell TCR-sequencing confirmed the clonal nature of tissue Tregs as the percentage of polyclonal TCRs decreased with the transition of Nfil3^-^ to Nfil3^+^ Tregs and was lowest in tissue-skewed Tregs also expressing *Klrg1* (24). Thus, tissue Tregs go through a two-step differentiation process, where they acquire a partial tissue Treg signature within the SLOs before seeding their target tissue in a TCR-dependent manner and adopt a terminally differentiated tissue-specific phenotype.

To ensure close proximity of Tregs and effector cells at the site of their activation, both are directed to peripheral tissues by tissue-specific molecules. These include CLA (cutaneous leucocyte-associated antigen) and the integrin a_4_b_7_, directing Tregs to the skin and the intestinal epithelium, respectively. CLA binds E-selectin which is uniquely enriched in the endothelium of the skin and up-regulated upon inflammation, while a_4_b_7_ binds to MadCAM-1, and together with the chemokine receptor CCR9, drives migration to the gastrointestinal-associated lymphoid tissue (GALT) (27). Interestingly, a considerable proportion of human circulating Tregs express CLA and CCR4, indicative of their ability to recirculate and migrate between skin and draining lymph nodes at steady-state (28–30). In contrast, very few circulating Tregs express the gut-homing receptors (29), suggesting that they represent a more resident subset. But what is driving the acquisition of the tissue-specific homing receptors? Several groups reported that tissue-specific dendritic cells (DCs) able to migrate to draining lymph nodes were involved in naïve T cell instruction both *in vitro* and *in vivo* (27, 31). Gut-tropic DCs are able to convert vitamin A captured from the diet into retinoic acid (RA), which induces the up-regulation of CCR9 and a_4_b_7_ in T cells (32). Similarly, IL-12 and TGF-β were suggested as drivers of CLA and other homing receptors in Tregs, such as CCR4, CCR10, P- and E-selectin ligands favoring migration to the skin (33, 34). DCs and soluble factors from distinct tissues can indeed induce expression of homing receptors specific for the same tissue in Tregs *in vitro.* RA promotes expression of gut-tropic homing receptors, and Tregs treated with RA can migrate to the gut to suppress DSS-induced colitis, an effect that was inhibited when treated with anti-a_4_b_7_ antibodies (17). IL-12 instead induces expression of skin-homing receptors enabling IL-12-cultured Tregs to infiltrate the inflamed foodpad (17). However, despite several tissue-specific chemokine receptor patterns on Tregs, some receptors are shared among different peripheral sites, indicating a certain redundancy (see **Table 1**).

**Table 1:** Homing receptors expressed on Tregs.

Tissue	Receptor	Context	Ref.
**Skin **	CLA, CCR6	The majority of circulating Tregs express these receptors and can infiltrate to the skin	(30)
	CCR4, CCR5	Tregs migrating to skin upon Leishmania-driven infection	(35)
	CCR6	Microbiota-driven CCL20 in hair follicles attracts Tregs to the neonatal skin	(36)
**Bone marrow **	CXCR4	Tregs sense SDF-1 to migrate and infiltrate, exit due to GM-CSF gradients	(37)
**Liver **	CLEVER-1	With the help of ICAM-1 and VCAM-1	(38)
**Heart **	CCR4	Observed in heart allograft experiments	(39)
**Peritoneum **	CCR4, CCR6, CXCR3	Tregs isolated from peritoneum after transfer and immunization	(18)
**CNS **	CCR6, CCR8	Migration towards CCL1, CCL20 and CCL22 produced by astrocytes	(40)
	LFA-1	In absence of Itga4, for Treg infiltration during CNS autoimmunity	(41)
**Gut **	a4b7, CCR9	RA drives the expression of CCR9 and a4b7, targeting Tregs to mesenteric LN	(17)
**SLO **	CCR7, CD62L	The acquisition of these receptors in the thymus drives Tregs to SLO	(7)
**Kidney **	CCR6	Stat3-driven CCR6 Tregs ameliorated disease score in pristane-induced lupus GN model	(42)
**Muscle **	CCR2	Expressed on Tregs infiltrating the injured muscle	(11)

Tregs up-regulate different surface receptors in order to reach the target tissue.

Interestingly, the site of antigen encounter not only has an impact on the homing capacity of Tregs, but also influences their function. In a peanut allergy model, different administration routes for the immunotherapy induced functionally distinct Tregs. These not only expressed different sets of chemokine receptors, but also differed in their expression and dependence on the suppressive mediators IL-10 and CTLA-4 as well as their longevity (43). Whether the site of Treg priming also has an impact on their non-canonical functions is still unclear. However, given that Tregs in peripheral tissues display an enhanced ability to e.g. produce the tissue protective factor amphiregulin (Areg), this will be important to address and may be an interesting therapeutic approach for pathologies marked by severe tissue-damage.

In addition to the site of priming, the chemokine expression pattern and function of Tregs are also affected by the cytokine environment present during an immune response. For instance, during type 1 immune responses, Tregs up-regulate the master transcription factor T-bet, which in turn drives CXCR3 expression, allowing them to migrate to the inflammatory sites (44–46). CXCR3 drives recruitment of T cells to sites of ongoing type 1 immune responses and also acts as a critical lung-homing receptor in respiratory viral infections (47). CXCL10, the ligand of CXCR3, is increased upon infection with different respiratory viruses, including Influenza, SARS-CoV-1 and SARS-CoV-2 (48, 49), allowing for an efficient recruitment of CXCR3^+^ Tregs to the lung and the site of the effector response. Treg-mediated suppression is strictly dependent on their localization in close proximity to the effector cells at the site of an ongoing immune response. Hence, blockade of any step of the migration process will interfere with Treg function. Nevertheless, as Treg and effector T cell require the same molecular interaction for their migration, any interference with Treg migration would also interfere with migration of effector T cells into the affected tissue.

Several strategies aimed at blocking effector T cell migration have been developed to treat autoimmune disorders. Examples include the monoclonal antibody Natalizumab targeting the α4β1 integrin (50) or the sphingosine-1-phosphate receptor modulator Fingolimod, which sequesters lymphocytes in lymph nodes (51). These therapies are successfully used in the treatment of autoimmune disorders such as Multiple Sclerosis (MS). While Natalizumab blocks infiltration of cells to peripheral sites and does not alter the Treg composition in blood (52), Fingolimod appears to sequester CD4^+^ Foxp3^-^ T cells in secondary lymphoid tissues more strongly than the Foxp3^+^ counterparts (53). This is due to the differential expression of S1PRs in the different T cell populations (54). Apart from this, signaling through S1P1R blocks the TGF-β pathway (55), which is crucial for Treg differentiation. Hence, treatment with Fingolimod not only removes potentially pathogenic lymphocytes from the circulation but may also interfere with the generation of Tregs. Another example on how defects in T cell migration could affect the Treg compartment is P-selectin ligand disfunction. PSGL-1^-/-^ mice have an enhanced susceptibility to EAE (experimental autoimmune encephalitis), the animal model for MS, due to the importance of the P-selectin ligand for Treg-mediated control of the autoimmune T cell response (56). In addition to these general migratory mechanisms, there are also some differences in the adhesion molecules required by different T cell subsets to enter peripheral tissues, e.g. the CNS. Th1 cells entail the integrin α4 to infiltrate the CNS whereas Th17 cells and Tregs depend on LFA-1 in the absence of integrin α4 (41, 57). As such, it seems likely that the functional outcome of T cell migration blockade depends on three key points: (i) the relative expression of the targeted molecule in effector T cells and Tregs, (ii) their dependence on that molecule or redundancy in targeting each population to a specific tissue and, iii) the relative importance of Treg recruitment for immune suppression *vs*. tissue protection and regeneration in the damaged organ (as discussed in detail in the next section). As such, the correct and timely localization of Tregs is a key event in controlling immune activation and maintaining tissue homeostasis. Tissue homing is essential to bring Tregs to the site of action, where they not only perform their immune suppressive functions, but also provide mediators for tissue protection.

## Treg Functions in Peripheral Tissues

**Figure 2:**
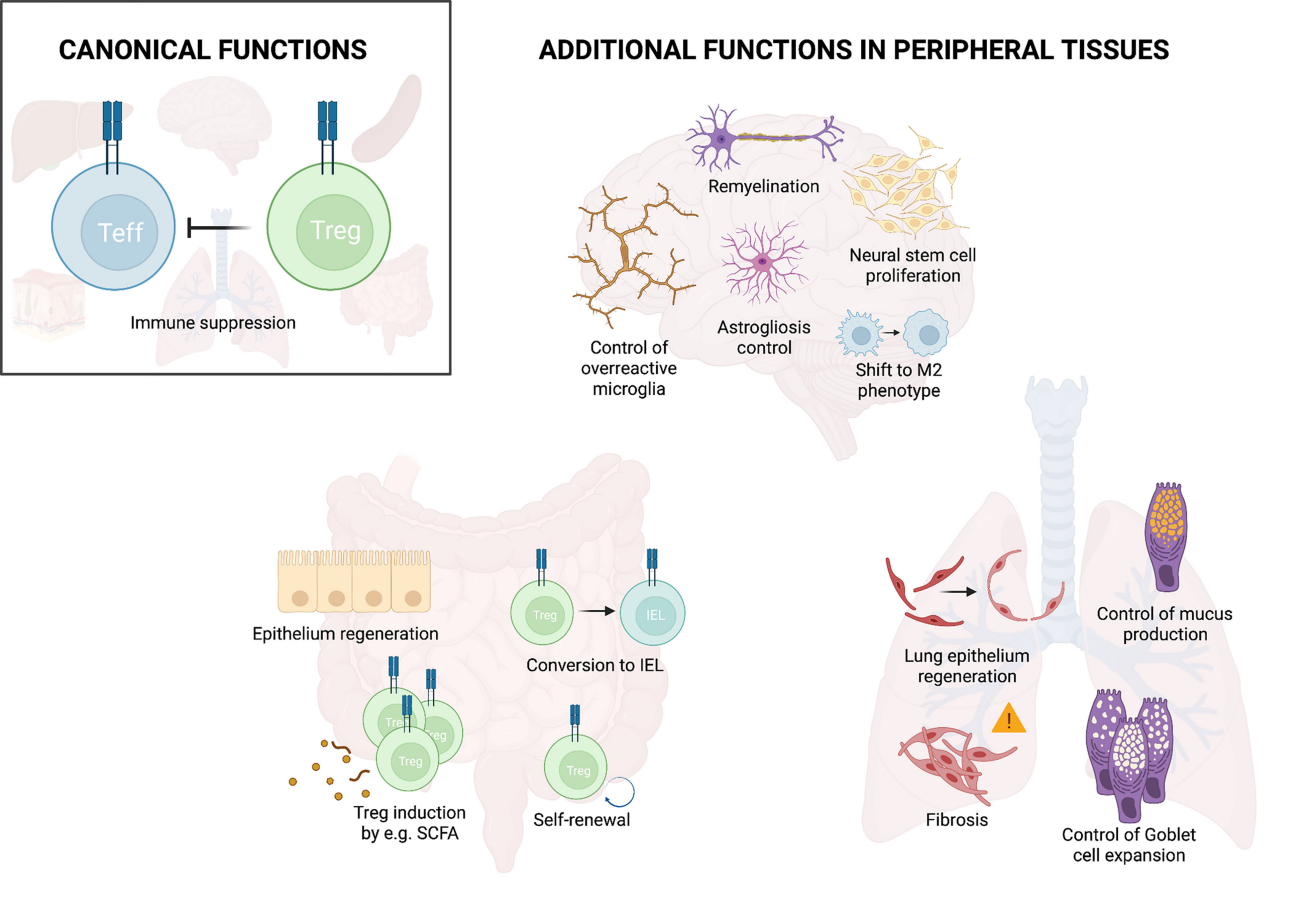
Treg functions in peripheral tissues. In addition to their main function of suppressing effector immunity, Tregs fulfill additional functions in peripheral organs. These include control of overreactive cells and promotion of tissue repair as illustrated for the brain, lung and intestine. Illustration created with BioRender.com.

Once in the tissue, Tregs can perform a wide range of functions. Their first and main role is to maintain homeostasis, to dampen excessive immune responses, and in this way avoid collateral tissue damage. Recent studies have revealed that in addition to this indirect role in tissue protection, Tregs also actively promote tissue maintenance, healing and regeneration (11, 58) (**Figure 2**). As such, Tregs e.g. promote hair regeneration by facilitating the function of hair follicle stem cells in the skin (59). Furthermore, Tregs can improve the formation and remodeling of scar tissue and reduce collagen degradation in the heart (60, 61). Similarly, Tregs interact with satellite cells to directly promote repair and regeneration after muscle injury and to counteract fibrosis (11, 58). This active role of Tregs in tissue regeneration has been observed in various tissues and the important pioneering work, describing their function in muscle, adipose tissue and skin, has been summarized in a number of excellent reviews [examples include (62, 63)]. Here, we will summarize our current understanding of this non-canonical function of Tregs in the intestine and the lung as examples of barrier sites as well as the CNS as an immune privileged site.

### Treg Features and Function in the Gut

Barrier sites are constantly exposed to external antigens and face the challenge of ensuring a potent immune response against pathogens while maintaining tolerance to commensals and harmless antigens. This challenge also extends to Tregs, which at these sites are largely specific for microbial antigens and to a large degree differentiate into Tregs at the barrier site itself (23, 64, 65).

**Figure 3:**
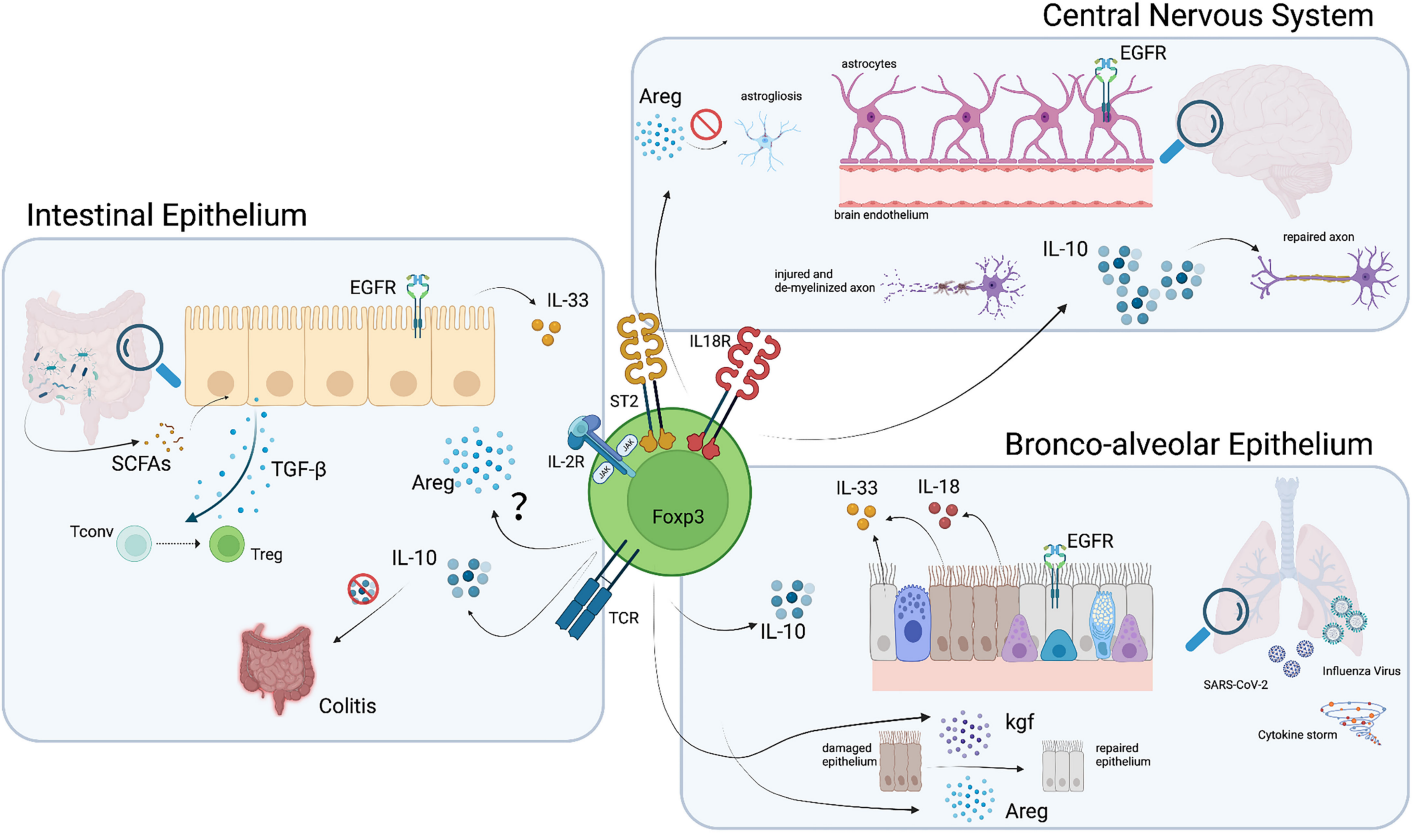
Pathways and molecules involved in Treg function in non-lymphoid organs. Tregs which home to the brain, lung and intestine acquire phenotypic characteristics that potentiate their function in these organs. Among these, the production of Areg and anti-inflammatory cytokines such as IL-10 and TGF-β, mediate Treg protective and reparative action. Illustration created with BioRender.com.

In the gut, the microbiota and the host tissue are separated by a physical barrier of intestinal epithelial cells (IECs). An extensive crosstalk exists between immune cells in the host tissue and IECs that conveys information from the gut lumen to the immune compartment and from immune cells to the IECs and the microbiota in the gut lumen (**Figure 3**). Foxp3^+^ Tregs that reside in the intestinal tissue are in their great majority induced from circulating conventional T cells, but there is also a fraction that originates in the thymus and homes to the intestine (66). In the lamina propria of the small intestine, a Treg niche has been characterized and some studies have addressed the possibility that these cells migrate to and are maintained in this organ independently from other immune system cues such as antigen encounter (67). *In vivo* studies with mice lacking peripheral MHC II expression have shown that, despite a reduction in the total CD4^+^ T cell count, adult MHC II KO mice have an increased frequency of Foxp3^+^ Tregs in the lamina propria (67). Despite the absence of MHC II, these Tregs show high CD44 expression, indicative of an activated phenotype. Furthermore, intestinal Tregs have a reduced expression of the IL-2 receptor subunit CD25 and, unlike other Tregs, their proliferation and maintenance is independent of IL-2 (15, 67). Instead, continued ICOS signaling is important for their maintenance (15). In addition, the alarmin IL-33, which is constitutively expressed by epithelial cells at barrier sites (68), acts as an intestinal survival factor for Tregs. The IL-33 receptor ST2 is enriched on colonic Tregs and IL-33 promotes expansion of these ST2^+^ Tregs (24, 69). Furthermore, IL-33 signaling also enhances TGF-β-mediated differentiation of Tregs and thus promotes Treg accumulation and maintenance in the inflamed gut tissue (69).

Interestingly, IL-33 not only plays an important role as a local survival factor for Tregs, but can also modulates their function by inducing Areg production (12). Areg promotes tissue regeneration by signaling through the epidermal growth factor receptor and multiple studies have demonstrated the ability of Tregs to produce Areg and promote tissue regeneration (11, 12, 70, 71). However, whether ST2^+^ Tregs in the gut also produce Areg and exert similar tissue protective and regenerative functions still remains to be determined. Besides Areg, which would directly act on barrier tissues, Tregs also produce immunomodulatory cytokines to promote homeostasis. One such anti-inflammatory cytokine is IL-10, which plays a crucial role in maintaining tolerance to commensal microbiota in the gut (5). IL-10 promotes tissue integrity by dampening effector immune responses against bacteria, viruses and parasites and hence dampening immuno-pathology. At the same time, it can also enable pathogen persistence, which may result in pathogen-induced pathology (72). Selective deficiency of IL-10 in Foxp3^+^ Tregs results in spontaneous colitis (73), suggesting that IL-10 is a key effector molecule for Treg-mediated tissue regeneration and protection. This likely not only holds true for the regulation of immune responses against microbiota, but also for those elicited upon infection (**Figure 3**). Overall, intestinal Tregs represent a highly specialized Treg subset that is essential for limiting inflammation through its secretion of anti-inflammatory cytokines. Furthermore, additional mediators of tissue protection, such as Areg, may enable them to actively promote tissue regeneration and thereby maintain tissue integrity.

### Influence of the Gut Microenvironment on Tregs

Non-canonical Treg functions are strictly associated with their localization and tissue adaptation as well as a fine-tuned crosstalk with the surrounding tissue. A good example of this is the gut, where continuous exposure to microbe- and food-derived antigens shapes the Treg niche to undergo a specialization driven by the tissue and food antigens present at this site. As such, intestinal Tregs can lose expression of Foxp3 and convert into non-inflammatory CD4^+^ intestinal epithelium lymphocytes (IELs) once they have migrated to the damaged epithelium (**Figure 2**). In this environment, they can co-operate with the remaining Foxp3^+^ Tregs, performing complementary roles in balancing intestinal inflammation and promoting an anti-inflammatory state (74). Notably, these local Tregs strictly depend on the intestinal microenvironment, i.e. microorganism-derived signals and defined components of the microbiota, for proper function and development (75). *In vitro* studies using a reporter cell line for TCR engagement showed that TCR activation in Tregs did not occur in the absence of microorganism-derived antigens (64). Nevertheless, germ-free mice have similar Treg frequencies and numbers as specific pathogen-free mice (76). In contrast, antigen-free mice harbor a dramatically reduced Treg population. Despite this dramatic reduction, antigen-free mice still maintained a small intestinal Treg population suggesting that not the entire Treg population in the gut is induced by local antigen (76). Besides providing antigen, commensal microorganisms in the gut also produce metabolites, such as the short-chain fatty acid (SCFA) butyrate, that promote Treg differentiation *in vivo* (77). A similar function has been assigned to food-derived fatty acids (78, 79). Dietary-derived SCFAs expand gut Tregs by suppressing the JNK and p38 signaling pathways (80). Conversely, dietary long-chain fatty acids (LCFAs) can induce Th1/Th17 differentiation, while decreasing SCFAs in the gut (80). LCFA treatment resulted in exacerbated autoimmune disease in the EAE model, which could be rescued by treatment with SCFAs *via* differentiation of lamina propria-derived Tregs (80). In addition to directly enhancing Treg differentiation, SCFAs produced by the microbiota enhance TGF-β1 production by IECs (81) (**Figure 3**). TGF-β1 then directly drives the conversion of conventional T cells into Foxp3^+^ Tregs in peripheral organs (82). Thus, SCFA-induced TGF-β1 production generates an environment that also indirectly promotes Treg induction *in vivo*. Specific symbionts in the mouse gut microbiome have been found to induce a subset of Tregs that co-expresses the transcription factor Rorγ together with Foxp3 (65, 83). This Treg subset plays an important role in controlling colonic inflammation. Intestinal Rorγ^+^ Tregs are mostly Helios^-^ and express high levels of IL-10, ICOS and CTLA-4 (65, 83), which mediate the strong suppressive capacity of these specialized Tregs. Nevertheless, whether these microbiota-induced Tregs only contribute to tissue homeostasis by limiting effector T cell activation or whether they also directly promote tissue protection and regeneration through release of tissue regenerative factors such as Areg still needs to be investigated. However, the studies we summarized here highlight the heterogeneity and plasticity of T cells and Tregs in the intestine as well as the fact that these Tregs are shaped by microbiota- and food-derived antigens and specialize to limit inflammation of the intestinal tissue and potentially participate in its renewal when damaged.

### Treg Functions in the Lung

The lung represents another non-lymphoid organ where Tregs have shown a strong involvement in tissue maintenance beyond the canonical immune homeostasis (**Figure 2**). Like the gut, the lung is highly exposed to external pathogens and microorganisms, which can potentially cause harm. Adaptive immune cells including Tregs colonize the respiratory tract and like in the gut, the Tregs have to maintain the delicate balance between allowing efficient immunity against pathogenic threats while maintaining tissue homeostasis and integrity.

Non-classical roles of Tregs in the lung tissue are still under investigation, but several independent studies have demonstrated their active involvement in lung epithelium regeneration (84, 85). Indeed, multiple studies have shown the ability of lung Tregs to produce Areg in response to IL-33 (11, 12, 70, 71). During lung inflammation, ST2^+^ Tregs exert tissue protective and regenerative functions through Areg production. Furthermore, Foxp3^+^ Tregs in the lung produce keratinocyte growth factor upon LPS stimulation. This factor, together with Areg, induces epithelial cell proliferation and subsequent tissue regeneration upon injury (85) (**Figure 3**).

Tregs have also been studied in acute lung injury (ALI), which leads to a progressive influx of CD3^+^ T cells including Tregs into the respiratory tract. Importantly, Treg influx into the alveolar compartment correlates with the transition from injury to resolution and Treg infusion promotes resolution of LPS-induced lung injury in mice, resulting in increased survival and reduced BAL protein and cell count (84). Interestingly, Tregs directly act on alveolar epithelial cells during the recovery phase of ALI by enhancing their proliferation and thereby accelerating resolution and lung tissue regeneration (86) (**Figure 3**). Furthermore, IL-10-producing Tregs play an important role in limiting type 2 inflammation in the lung. In an OVA-induced lung inflammation model, lack of Treg-derived IL-10 results in exacerbated immune pathology characterized by increased mucus production, Goblet cell expansion, edema, as well as lymphocyte and eosinophil infiltration (73). Nevertheless, Tregs can also have a negative impact on the lung tissue by promoting fibrosis through secretion of IL-10 and TGF-β (87). This once again highlights the need for a fine-tuned balance to allow prevention of immune-pathology while promoting tissue regeneration.

In addition to limiting lung pathology in settings of sterile inflammation, Tregs also play an important role in mitigating lung damage upon infection. Like upon injury, lung Tregs release Areg in response to Influenza Virus infection, thereby counteracting pathogen-induced as well as immune-mediated pathology (12). Importantly, this tissue protective effect is independent of the immune suppressive function of Tregs and their classical regulatory role (12). In addition, Tregs have been addressed in the context of SARS-CoV-2 infection and several studies revealed that in patients with severe COVID-19, marked by potent cytokine storm, Tregs might be beneficial for the lung environment, dampening the pro-inflammatory cues and restoring tissue integrity (88) (**Figure 3**). On the other hand, excessive immune regulation by Tregs may hamper an effective immune response against SARS-CoV-2 and thereby exacerbate disease severity (89). Of note, Tregs in COVID patients are phenotypically distinct from Tregs observed upon Influenza Virus and Respiratory Syncytial Virus infection (89, 90). As mentioned earlier, Tregs up-regulate CXCR3 in response to the infection, which directs them to the lung as the site of an ongoing immune response (44). While the recruitment of T-bet-expressing CXCR3^+^ Tregs to the site of type 1 immune responses is essential for the efficient control of these responses (44, 45), it is still unknown whether the same Treg subset also exerts the tissue protective functions observed upon infection (12). Understanding the exact functions of each Treg population will be particularly important in infectious settings in which immune-mediated damage correlates with disease severity, such as Influenza or COVID-19 (91, 92). In conclusion, Treg function may contribute to pathology if they excessively limit immunity to infection. Nevertheless, Tregs also play an important role in limiting sterile, immune-mediated, or pathogen-induced tissue damage by shaping the immune environment but also by acting directly on epithelial cells.

### Long-Lasting Treg Responses in Peripheral Tissues

As discussed, the main function of Tregs at barrier sites is to maintain tolerance where continuous environmental cues are faced. In addition, accumulating evidence for a memory phenotype of Tregs at these sites has emerged. A clear phenotypical characterization of a Treg memory subset is still lacking but, in the past years, evidence of the persistence of memory-like Tregs in peripheral lymphoid and non-lymphoid tissues has arisen, especially at barrier sites (93). Functional Treg memory was assessed in the lung, where adoptive transfer of memory but not naïve Tregs from SLOs attenuated lung pathology following Influenza infection (94). Similarly, memory Tregs capable of suppressing a secondary inflammatory response upon antigen re-encounter have been observed in pregnancy. In this context, fetal antigen-specific Tregs persist in SLOs even beyond the pregnancy. These Tregs re-expand following re-encounter with the same antigen in a subsequent pregnancy and maintain fetal tolerance (95). However, whether fetal-antigen-specific Tregs are also maintained as a tissue-resident memory population or are only present in SLOs still needs to be determined. Guo and colleagues defined a subset of CD4^+^CD25^+^Foxp3^+^ T cells in the lamina propria which has effector/memory-like properties and is characterized by low expression of CD62L and CD45RB while expressing CD44 and the chemokine receptors CCR4 and CCR9, which are not exclusively expressed on memory cells but mark circulating and tissue-infiltrating lymphocytes (96) (**Figure 1**). However, whether these memory Tregs are maintained in the tissue and represent a truly tissue-resident population is unclear. Moreover, a mouse model allowing for the inducible expression of a self-antigen in the skin clearly showed that a memory Treg population is maintained following antigen clearance and contraction of the effector cytokine response (97). A follow-up study then also identified the corresponding memory Treg subset in humans (98). Moreover, IL-2 has been shown not to be required for the generation of memory Tregs in the skin, while IL-7 is essential for maintaining them at steady state in this organ (99). While these studies showed that memory Tregs persist in the skin, it is still unclear whether they are replenished from SLOs or whether they represent a truly tissue-resident memory population that is maintained locally.

The existence of memory Tregs has been explored for decades now, but a distinct characterization of their phenotype, maintenance, and mechanism of action in the peripheral tissue is still missing and will require further investigation. Nevertheless, as highlighted above, accumulating data suggest that Treg memory may play a key role in tissue protection in peripheral organs. However, as memory Tregs share phenotypical characteristics and functional features with effector Tregs, further studies are needed to clearly define their markers to allow for a clear identification of these cells. Furthermore, it will be important to determine whether memory Tregs residing in peripheral tissues can recirculate or whether they are tightly restricted to the tissues and represent a tissue-resident population.

### Role of Tregs in the Central Nervous System

In contrast to barrier tissues, the central nervous system (CNS) is shielded from external antigens and holds immune privilege, conferring protection from potential damage of the peripheral immune system to this site. As such, Treg numbers in brain tissue at steady state are very low, under 100 cells in most of the cases. Despite this low count, Tregs are present and show a similar phenotype as those observed at barrier sites in association with tissue repair (**Figure 2**). Like barrier Tregs, CNS Tregs express the IL-33 receptor ST2. In addition, they also express the serotonin receptor as a tissue specific receptor and can survive and proliferate in absence of IL-2 upon IL-33 and serotonin sensing (40).

While the CNS is well guarded from immune-mediated insults, some pathogens may exploit the immune privilege and target the CNS to expand. When that happens, Tregs (and other T cells) can infiltrate the brain upon inflammation, following the CCL1 and CCL20 gradient produced by astrocytes (40). Effector Th1 cells and memory cells may enter the CNS through the blood-brain barrier, while Tregs and Th17 can reach the CNS through the choroid plexus (100), emphasizing a difference between these subsets of CD4^+^ T cells. This variation in the entry site could influence their final location, and may therefore influence their main function and purpose as well (101, 102). After infiltration, the main role of Tregs is to suppress ongoing immune responses and dampen inflammation to protect neural integrity. Nevertheless, other functions have been attributed to them, making Tregs a key protective cell population of the nervous system (**Figure 2**). To start with, Tregs can control overreactive microglia, either by reducing their activation and cytokine secretion, by decreasing their production of neurotoxic factors (e.g. nitric oxide, NO, and inducible nitric oxide synthase, iNOS) through driving a shift to the anti-inflammatory M2 phenotype or by inducing their apoptosis (103–105). As NO is one of the major causes of neuronal apoptosis, this role of Tregs is crucial. On the other hand, astrogliosis (or abnormal astrocyte growth) can also be dampened by Tregs through the release of Areg (40) (**Figure 3**). Moreover, Areg can potentiate neural stem cell proliferation (106). Again, the fact that Areg also acts in immune privileged tissues highlights the importance of Treg-derived Areg in tissue protection. During sterile CNS damage as it may occur during a stroke, Tregs can contribute to the resolution of the acute phase either directly or by promoting macrophages to adopt a M2 phenotype (107, 108). Moreover, Treg depletion during EAE impairs remyelination, a property that can be regained upon Treg transfer (109), and explains why a diet restricted in SCFAs reduces axonal damage through Treg induction, as discussed in detail in an earlier section (80). In addition, the fact that Treg numbers do not decrease during the remission phase, as it occurs for CD4^+^ effector T cells, highlights that the role of Tregs in tissue protection continues beyond the inflammatory phase (110).

Despite their important role in maintaining CNS homeostasis, Tregs are a double-edged sword when it comes to CNS infection: their proliferation and activation can dampen effector immune responses, allowing e.g. viruses to spread in the CNS (111). On the other hand, Treg depletion can allow excessive infiltration and activation of effector T cells in the brain, causing strong immune pathology and damage (112). Furthermore, Tregs have been observed to directly act on infected innate immune cells and drive their apoptosis *via* the caspase-3 and the perforin/granzyme B pathway (61). Hence, Tregs can be involved in viral clearance from the brain, as observed in Human Immunodeficiency Virus (HIV) infection (113). Moreover, during HIV infection, brain-infiltrating Tregs can potentially promote the conversion of M1 to M2 macrophages, which adopt an anti-inflammatory phenotype, and thereby protect the morphology of the dendrites (114). IL-10 secretion by Tregs has also been associated with neuroprotection upon viral infection, independent from immune suppression (115). In the case of Herpesvirus, Tregs from acute murine cytomegalovirus (MCMV)-infected animals prevent neural damage by restraining microgliosis and astrogliosis and can avoid hippocampal injury and cognitive impairment (116). Interestingly, Tregs also promote memory T cell formation in the CNS after MCMV-infection (117). Therefore, besides acting on effector T cells to suppress collateral T cell damage, Tregs can actively support formation of immunological memory. Treg depletion prior infection with the model coronavirus MHV (mouse hepatitis virus) did not interfere with T cell infiltration but led to increased apoptosis of neurons and myelin loss (118), suggesting that rather than being mainly involved in T cell suppression, CNS Tregs have a central role in tissue protection after infection. In line with this, it is tempting to hypothesize that brain Tregs could ameliorate the neurological symptoms derived from COVID-19 infection (119).

All in all, the potential beneficial roles of Tregs in CNS will depend on the circumstances. Most studies focus on the role of Tregs in limiting effector T cell activation and immunopathology, while little is known about their direct impact on neural cells. Regarding viral infections, we understand that Tregs hamper viral clearance in the periphery and may thereby facilitate virus infiltration in the CNS. Nevertheless, depending on the type of virus, Tregs can also promote anti-viral effects and may play a favorable role, particularly during the late stage of encephalic disease, when their tissue protective functions take the lead.

## Conclusions

Tregs are critical for maintaining tissue homeostasis both by inhibiting excessive immune activation and by directly interacting with the cellular network in the tissue niche to promote tissue renewal and repair. While a core set of features is shared between Tregs in lymphoid organs and peripheral tissues, many non-lymphoid tissues harbor phenotypically distinct Treg subsets with unique functions that we are only beginning to understand. Nevertheless, some general concepts have started emerging.

First, Treg activity is strictly dependent on their localization at a given tissue site, which is controlled by their specificity and site of priming, their migration to a specific tissue, and the availability of growth and survival factors within that tissue. As a part of their adaptation to non-lymphoid tissues, Tregs become less dependent on IL-2 and are instead maintained by other cytokines such as IL-7 and IL-33 or by tissue specific factors like serotonin (40, 69, 99). Furthermore, the milieu and cellular network present in different tissues, such as microbiota derived factors and abundance of TGF-β at barrier sites, promote Treg differentiation and thereby control the amount and functional impact of Tregs at that site (65, 75, 77).

Second, classical immune regulation by Tregs at peripheral sites and their direct tissue protective effects rely on distinct functional programs. While immune suppression involving co-inhibitory receptors and the modulation of APCs come into play during immune suppression in lymphoid tissues, suppressive cytokines like IL-10 and TGF-β are important functional mediators in both lymphoid and non-lymphoid organs (5). In addition, Tregs in peripheral tissues produce Areg - and likely also other factors still to be identified - that directly act on tissue cells to regulate their regeneration and function (11). Whether the classical immune suppressive and the regenerative function of Tregs are carried out by the same Treg population still needs to be determined. Similarly, the conditions that allow for Tregs to acquire this regenerative capacity, which may include the site and conditions present during their initial priming or simply the presence of the right stimuli at the tissue site, remain unclear. A better understanding of these processes will potentially allow for the manipulation of Tregs in therapeutic approaches targeting their regenerative role.

## Author Contributions

All authors wrote and revised the manuscript. All authors contributed to the article and approved the submitted version.

## Funding

This work was supported by the European Research Council (grant No. 677200 to NJ).

## Conflict of Interest

The authors declare that the research was conducted in the absence of any commercial or financial relationships that could be construed as a potential conflict of interest.

## Publisher’s Note

All claims expressed in this article are solely those of the authors and do not necessarily represent those of their affiliated organizations, or those of the publisher, the editors and the reviewers. Any product that may be evaluated in this article, or claim that may be made by its manufacturer, is not guaranteed or endorsed by the publisher.
